# Supplementary material for: A Next-Generation Adenoviral Vaccine Elicits Mucosal and Systemic Immunogenicity and Reduces Viral Shedding after SARS-CoV-2 Challenge in Nonhuman Primates
Source: Vaccines (Basel). 2024 Jan 27;12(2):132. doi: 10.3390/vaccines12020132 (PMC10893453; doi:10.3390/vaccines12020132)
Supplement: Supplementary file 1 [file vaccines-12-00132-s001.zip › vaccines-2831518-supplementary.pdf]

Table S1. African Green Monkey immunization group assignments and baseline characteristics.

Immunization Group	Identification Number	Sex	Body Weight (kg)
Control	659706	Female	3.45
	659585	Female	4.35
	659606	Male	4.90
	659247	Male	6.55
	659845	Male	6.85
ED88	659533	Female	3.80
	659798	Female	4.45
	659366	Female	4.75
	200943	Male	5.55
	659812	Male	6.40
	659377	Male	7.05
ED90	659946	Female	3.95
	659397	Female	4.20
	658193	Female	4.45
	659007	Male	5.55
	659382	Male	6.00
	659460	Male	7.05
Protein + ED94	658739	Female	3.95
	658186	Female	4.25
	659333	Male	5.80
	659370	Male	7.35
ED94	658207	Female	4.00
	659187	Female	4.30
	658139	Female	4.95
	659449	Male	5.60
	658727	Male	6.45
	659536	Male	5.58

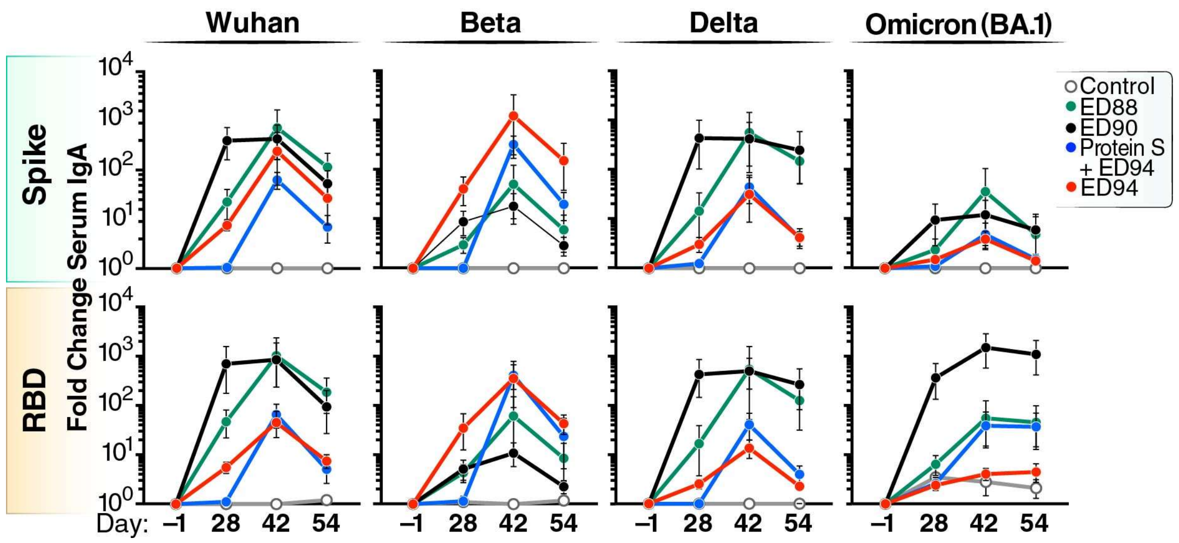

Figure S1. Mucosal immunization generates cross-reactive serum IgA responses. Serum spike specific IgA was quantified by MSD against Wuhan, Beta, Delta, and Omicron (BA.1) variants on days -1, 28, 42 and 54. Data include vehicle control animals (open white circles, n = 5), ED88 (green circles, n = 6), ED90 (black circles, n = 6), intramuscular delivery of spike protein followed with ED94 boost (blue circles, n = 4), and ED94 (red circles, n = 6). Data expressed as fold change from baseline at day -1; top row full length trimerized spike, bottom row RBD. Abbreviations: IgA, immunoglobulin A; RBD, receptor binding domain.

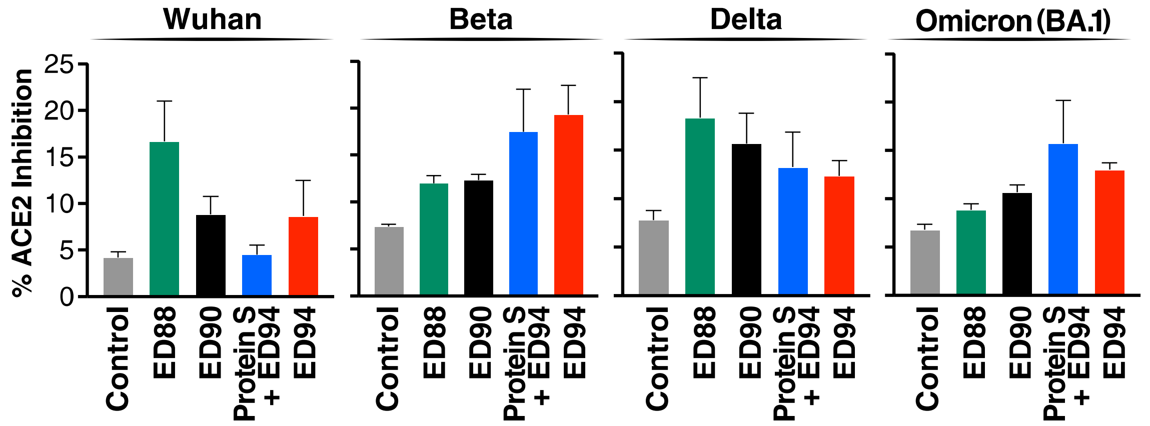

Figure S2. Mucosal immunization enhances neutralizing antibodies in the lower airways. Neutralizing antibody activity against the RBD portions of Wuhan, Beta, Delta, and Omicron proteins in BALF by sVNT at day 54 prior to challenge. Data include vehicle control animals (grey bars, n = 5) and vaccinated groups ED88 (green bars, n = 6), ED90 (black bars, n = 6), intramuscular delivery of spike protein followed with ED94 boost (blue bars, n = 4), and ED94 (red bars, n = 6). Data expressed as mean ±SEM. Abbreviations: ACE2, angiotensin-converting enzyme-2; BALF, bronchoalveolar lavage fluid; RBD, receptor binding domain; SEM, standard error of the mean; sVNT, surrogate virus neutralization test.
